# Women’s perceptions of factors needed to encourage a culture of public breastfeeding: a cross-sectional study in Sweden, Ireland and Australia

**DOI:** 10.1186/s13006-023-00583-z

**Published:** 2023-09-01

**Authors:** Charlotta Dykes, Pernilla Ny, Yvonne L. Hauck, Lesley Kuliukas, Louise Gallagher, Vivienne Brady, Christine Rubertsson

**Affiliations:** 1 Department of Health Sciences, Medical Faculty, Lund University, Sölvegatan 19 223 62, Lund, Sweden; 2 Department of Pediatric Neurology, Skane University Hospital, Lasarettsgatan 48 222 41, Lund, Sweden; 3 School of Nursing, Curtin University, Kent Street, Bentley, WA 6102 Australia; 4 School of Nursing and Midwifery, Trinity College Dublin, The Gas Building, 24 D’Olier Street, Dublin, D02 T283 Ireland; 5 Department of Obstetrics and Gynecology, Skane University Hospital, Jan Waldenströmsgata 47 214 28, Malmö, Sweden

**Keywords:** Public breastfeeding, Breastfeeding culture, Experiences, Mothers, Women’s rights, Children’s rights, Public knowledge, Social norms, Attitudes

## Abstract

**Background:**

Breastfeeding in the public sphere is known to be experienced as a problem for many women. It has been shown to arouse negative feelings among the public, depending on the attitude of those in the immediate surroundings. This contributes to the fact that many women hesitate to breastfeed in public and prepare themselves for potential adverse comments.

**Methods:**

An online survey was used for an international cross-sectional study including women living in Sweden (n = 1252), Australia (n = 7602) and Ireland (n = 1597). Women who had breastfed within the previous two years were invited to participate through Facebook. One key open-ended question was presented, inviting women to respond to: “What do you think is important or needed to encourage a breastfeeding culture where breastfeeding in public is seen as normal?” During 2018, data were collected during a four-week period. A thematic analysis of women’s responses was conducted separately in each country and then comparison and negotiation occurred once similarities between themes and subthemes were confirmed. Frequencies of subthemes were then determined and compared between the three countries.

**Results:**

Seven subthemes developed from the data; ‘Make breastfeeding visible in society’; ‘Healthcare professionals support and knowledge regarding breastfeeding’; ‘Education of the public’; ‘Inviting environment’; ‘Zero tolerance to other’s unwanted opinions’; ‘Focusing on the needs and rights of the breastfeeding dyad’; and ‘Desexualize breastfeeding and women’s’ bodies in society’. Subthemes were integrated under two themes; ‘Active supportive interventions needed for breastfeeding’ and ‘The obvious right of breastfeeding women and children to take a seat in the public sphere’.

**Conclusion:**

The common experience that exists today regarding public breastfeeding requires change towards normalization. Further collaborative research is recommended to meet the expressed requirements from women who wish to breastfeed in public.

## Background

Challenges with breastfeeding in public continue to pose a threat to the World Health Organization’s (WHO) recommendation that infants be exclusively breastfed for the first six months of life [1]. In consultation with the United Nations Children’s Fund, the WHO has developed a breastfeeding strategy where the importance of not giving newborns supplements other than breast milk is emphasized, encouraging women to breastfeed on demand [1, 2]. The Convention on the Rights of the Child ensures that all groups in society, especially parents, should be educated about child health care, benefits of breastfeeding, nutrition and clean environments and that members of the convention must take appropriate and effective strategies to reduce societal expectations that may be detrimental to children’s health [3].

The protection of breastfeeding in public has been considered within the context of three developed countries: Australia, Ireland and Sweden, chosen for their differing breastfeeding initiation and prevalence rates. An Australian federal Sex Discrimination Act was introduced in 1984, making it illegal in every district and territory to discriminate against a person on the grounds of breastfeeding [4]. This Act is comparable to The Equal Status Act and Act on Gender from Ireland [5]. Similarly, the Swedish government has focused on its equality policy subgoals to address gender-specific conditions more relevant to women such as breastfeeding rights and the risks that may occur when women perceive they are being compromised when undertaking this important activity in public [6]. Although these initiatives have attempted to protect women breastfeeding in public, current breastfeeding rates reflect differences across these countries. The breastfeeding initiation rates are 96% in Australia and 93% in Sweden whereas in Ireland, the rate is 62%. At the age of six months the frequency of breastfeeding is 60% (Australia), 72% (Sweden) [7–9] and 26–29% (Ireland) [10] which is not in accordance with the WHO recommendations [1]. In comparison, a previous study regarding exclusive breastfeeding in infants aged less than six months showed that 37% were being exclusively breastfed in middle- and low-income countries. In countries with higher incomes, the duration was even lower [11].

Internationally, lack of support and critical attitudes have influenced women’s perceptions of breastfeeding in the public sphere [12]. Women have confirmed that breastfeeding in public is challenging and they have also shared what would be helpful such as having comfortable seating, seeing other mothers breastfeed, knowledge of mother’s and children’s rights, understanding and acceptance from others, confidence, and having breastfeeding overtly welcomed [13]. Although interventions have been implemented internationally to improve and normalize public breastfeeding, evaluation around the impacts of these interventions has been recommended [14, 15].

The Swedish Centre of Excellence in Women ´s Health indicated that attitudes to breastfeeding are generally positive but in the context of public breastfeeding attitudes vary. For example, three out of ten respondents agreed that bottle feeding is more accepted than breastfeeding in public. Men, students and younger respondents tended to be more concerned about breastfeeding in public than other respondents [16]. Sexualization of the female breast, where the breast often is portrayed sexually in social media, films, television, newspapers, and advertising may be contributing to these views [17].

More evidence is needed regarding breastfeeding women’s needs when breastfeeding in public, in order to influence public attitudes and societal actions to better support women’s decisions when to breastfeed in a public sphere. Therefore, the aim of this manuscript is to report on what breastfeeding women in Australia, Ireland and Sweden perceive is needed and important to encourage a culture of public breastfeeding.

## Method

An online survey was used for an international cross-sectional study in 2018 that included women living in Sweden, Australia and Ireland. Women who were currently breastfeeding or had breastfed within the previous two years were invited to participate through social media (Facebook). User friendly platforms that allowed completion on a mobile phone were used for the short online survey in each country including Qualtrics [18] in Australia and SurveyMonkey [19] in Ireland and Sweden. A poster with a link to the online survey was circulated for one month in each country. The translated poster in Swedish was included in several Facebook pages with parental interest such as home parent’s network and a public breastfeeding page. In Australia the poster was posted on a maternity consumer Facebook page that encouraged women to share with other women. In Ireland a breastfeeding support on Facebook group was used for the recruitment.

The initial screen of the online survey contained an information letter which incorporated the inclusion criteria. Women had to confirm that they were living in Sweden, Australia or Ireland and meet the inclusion criteria of currently breastfeeding or having breastfed within the previous two years. The final compulsory element asked women to confirm their consent to participate prior to being able to access the survey. The data collection was based on a survey including both standardized and study specific questions as well as nine open-ended questions. The current study presents results from one question: “What do you think is important or needed to encourage a breastfeeding culture where breastfeeding in public is seen as normal?” followed by a request to rank their own three most important suggestions. Our first publication addressed the objective of exploring and comparing what women do when faced with having to breastfeed in the presence of someone they are uncomfortable with and what women think is helpful and challenging when considering whether to breastfeed in public [13]. The final publication will report on how often and where the women breastfed in public, their comfort levels in front of different audiences, the support of their partner and how often they saw other mothers breastfeed.

The Demographic data in this study included maternal age, level of education, number of children, number of children ever breastfed and whether they were still breastfeeding their youngest child at the time they were completing the online survey. The women were invited to rank the perceived importance of their suggestions as one, two or three in relation to encouraging a breastfeeding culture and data were analysed with descriptive and comparative analysis to identify probable differences between countries using SPSS. During 2018, data were collected in March in Australia, April in Ireland and in December in Sweden; over a four-week period.

Responses to the open-ended question regarding what women perceived was needed to encourage a breastfeeding culture offered a substantial amount of data, although not in depth, with no word limitations in the online platforms for each country. The data were analysed using analysis guided by Braun and Clarke’s steps [20] where themes and subthemes were developed. Qualitative descriptive approaches that utilise content analysis or thematic analysis are acknowledged for their value in lower-level interpretation. In this stance, content analysis was selected to allow for description at a surface level for similar shared perceptions as captured in the women’s words from extensive textual information, to categorize patterns of words into themes and to count their frequency [21, 22]. As a first step, text in the open-ended question was read through by the researchers in each country several times, to give a clear picture of its content. The content was then discussed among the researchers to reach a deeper understanding. Step two, which started inductively involved systematically identifying codes from the entire data material to ensure that all information in the material was made visible. In step three, subthemes were formed based on the generated codes, in a moving process that involved both division and merging of codes. According to the method [20] that describes the inductive approach to the material, the subthemes developed from the bottom and up, driven by the content of the data and matching data closely to give “a voice” to the experiences. The fourth step meant that the subthemes that developed were discussed between the international researchers by summarizing and substantiating its content with representative quotations. Based on the above process, the researchers were able, as the fifth step, to highlight two clearly prominent themes which represented the content of the data. The same content analysis process was simultaneously conducted separately by two researchers in Australia and two in Ireland until no new findings were extracted from the data and similarity between the themes and subthemes was confirmed across countries. Participant quotes presented in the results to illustrate the themes and subthemes were identified by country and participant number.

Three separate SPSS databases were developed, one for each country as data were extracted from the survey platforms into SPSS files. Once the themes and the subthemes were confirmed following the thematic analysis, the data responses for each woman were matched to a subtheme and their ranking as ‘number one most important factor’ is presented in Table 1. The calculation was made of the number of times a matched subtheme returned in the women’s ranking. The women’s suggestions of their second and third most important factors were included in the thematic analysis but not in any calculation. This process allowed for frequencies being generated according to the importance perceived by women to encourage a breastfeeding culture in each country. As Australian responses were over four times (n = 7602) those from Sweden (n = 1252) and Ireland (n = 1597), every fourth response was systematically extracted in order to avoid selection bias and entered manually into a separate SPSS file with the recorded ranking of first, second or third for importance to generate frequencies of Australian women (n = 1900) to construct a comparative sample size to the Swedish and Irish samples. However, all Australian responses were incorporated in the content analysis employed by the Australian investigators to determine the subthemes and themes.

**Table 1:** Demographic characteristics of women offering feedback on what is needed to encourage a breastfeeding culture

Demographic characteristics	Women living in Australia n = 7602	Women living in Ireland n = 1597	Women living in Sweden n = 1252
**Maternal age**	32.5 mean	34.9 mean	32.7 mean
	Range 17 to 53 (SD 4.76)	Range 18 to 49 (SD 4.1)	Range 18 to 57 (SD 5.0)
**Level of education**			
Higher degree	2229 (29.3%)	821 (51.4%)	698 (55.8%)
No higher degree	5373 (70.7%)	761 (47.6%)	554 (44.3%)
**Number of children**			
1 child	3460 (45.5%)	672 (42.1%)	552 (44.1%)
2 or more	4142 (54.5%)	894 (56.1%)	612 (48.9%)
**Number of children ever breastfed**			
1 child	3597 (47.3%)	727(45.5%)	578 (46.2%)
2 or more	4005 (52.7%)	884 (55.4%)	587 (46.9%)
**Currently breastfeeding**			
Yes	5388 (70.9%)	1133 (70.9%)	800 (63.9%)
No	2214 (29.1%)	464 (29.1%)	452 (36.1%)

NOTE: Numbers may not add up to sample numbers as not all women responded to each item

## Results

The demographic data represent the women included in the study to describe the sample from each country, where in total 10,451 women participated in the study (Sweden n = 1252, Australia n = 7602 and Ireland n = 1597) (Table 2). The average age among the participants from the different countries was similar, with the Australian women being the youngest (mean 32.5 years), followed by the Swedish women (mean 32.7 years) and the women from Ireland (mean 34.9 years). The majority of the women in all three countries had some form of university education, with the Swedish and Irish participants being at a slightly higher level of education. Data regarding the number of children each woman gave birth to was very consistent with most participants giving birth to one child: Sweden 44.1%, Australia 45.5%, and Ireland 42.1%. Most of the participants had also breastfed one child and were actively breastfeeding when the data collection was carried out.

**Table 2:** Overview of themes and subthemes

**Theme**	**Subthemes**
Active support interventions needed for breastfeeding	Make breastfeeding visible in society
	Healthcare professionals support and knowledge regarding breastfeeding
	Education of the public
	Inviting environment
**Theme**	**Subthemes**
The obvious right of breastfeeding women and children to take a seat in the public sphere	Zero tolerance for others’ unwanted opinions
	Focus on the needs and rights of the breastfeeding dyad
	Desexualize breastfeeding and women’s bodies in society

The results are presented under the themes and corresponding subthemes. Table 1 provides an overview of themes and subthemes. A comparison of the number one most important subtheme between the three countries is offered as noted in Table 3.

**Table 3:** Subtheme frequencies for what the women ranked as number one most important and needed to encourage a breastfeeding culture

**Themes and subthemes**	Women living in Australia (n = 1900) n (%)	Women living in Ireland (n = 1597) n (%)	Women living in Sweden (n = 1252) n (%)
**Theme - Active supportive interventions needed for breastfeeding**			
*Subthemes*			
Make breastfeeding visible in society	550 (29%)	710 (41%)	594 (47%)
Healthcare professionals support and knowledge regarding breastfeeding	88 (5%)	164 (9%)	126 (10%)
Education of the public	544 (28%)	532 (31%)	185 (15%)
Inviting environment	266 (14%)	191 (11%)	134 (11%)
**Theme – The obvious right of breastfeeding women and children to take a seat in the public sphere**			
***Subthemes***			
Zero tolerance to others’ unwanted opinions	109 (6%)	25 (1%)	48 (4%)
Focusing on the needs and rights of the breastfeeding dyad	162 (8%)	91 (6%)	75 (6%)
Desexualize breastfeeding and women’s bodies in society	181 (9%)	32 (2%)	89 (7%)

### Theme: active supportive interventions needed for breastfeeding

The subthemes that are captured under this theme are ‘Make breastfeeding visible in society’; ‘Healthcare professionals support and knowledge regarding breastfeeding’; ‘Education of the public’ and ‘Inviting environment’.

#### Subtheme

‘Make breastfeeding visible in society’

The subtheme ‘Make breastfeeding visible in society’ includes comments regarding making breastfeeding a ‘normal’ part of life that was commonly seen in society. The women wanted breastfeeding to be viewed as something women chose to do to nourish and be close to their children. They aspired to a society where no one else sees breastfeeding as unacceptable. The desire that public breastfeeding be seen as visible emerged, as they felt supported when seeing other breastfeeding women:

People should feed as and when is needed. If we all treat it as normal it will be more readily accepted. When a taboo is made of it then it becomes a problem. [Aus; 4]

Many women shared how it would be useful if breastfeeding in public were a common act where breastfeeding women felt comfortable to be observed in public. The reality of seeing breastfeeding mothers in the public space should make it the norm, regardless of whether the child was an infant or toddler. They also suggested that it would be normalizing if more famous people were to support, speak openly and express positive views about breastfeeding. Positive portrayals of breastfeeding in advertising on mainstream media, social media, in films, art and newspapers was mentioned as helpful factors to make breastfeeding visible in society and that celebrities could talk more openly and positively about public breastfeeding. Respondents also expressed a need for promoting breastfeeding on all levels in the social and political hierarchy to reflect a society which promotes and legitimizes public breastfeeding.

#### Subtheme

‘Healthcare professionals’ support and knowledge regarding breastfeeding’.

Our participants wanted timely, concrete, and updated information from health care professionals (HCP) regarding breastfeeding and breastfeeding in public during the antenatal and postpartum periods, as they noted that the information given was often insufficient and out of date. Adequate information and encouragement from HCP could contribute to a greater feeling of comfort for the women in a public breastfeeding situation, which in turn, could increase the occurrence of and influence on perceptions of breastfeeding in society.

*That maternity care (antenatal classes, maternity ward etc.) encourage to breastfeed in public.* [Swe; 462]

Additional information was requested about the importance of breastfeeding, with regard to nutrition as well as attachment and bonding. Furthermore, it was communicated that the information from HCP regarding the benefits of breastmilk compared to commercial milk formula assisted in making the decision and commitment to breastfeed.

#### Subtheme

‘Education of the public’.

The women shared how universal breastfeeding education is an important aspect of normalizing breastfeeding in public. Providing accurate information to the public in general, and early education in schools were considered to be important ways to shift cultural attitudes. Women suggested that this information should begin as early as possible in the school system and should be reinforced at recurring intervals. They also voiced a need for education to convey knowledge about breastfeeding health benefits for the child such as optimal and individualized nutrition; antibody transfer that promotes immunity; and that breastfeeding is important, natural and promotes the child’s sense of security. Further knowledge among the general public was considered important to better understanding and acceptance towards public breastfeeding.

*Educate children, teenagers, men and older people that it’s natural and best for baby. . and it’s what breasts are for.* [Ire; 106]

The women reported that education can promote knowledge and decrease the likelihood of being harassed with negative comments while breastfeeding in public. The women also insisted that information needed to be given to people in general that breastfeeding women need to respond to their child’s signals and that breastfeeding is recommended.

#### Subtheme

‘Inviting environment’.

Clearly marked out spaces were requested, with signs welcoming breastfeeding mothers to contribute to a more comfortable atmosphere. The wish for a clarification that breastfeeding was welcome was emphasized, not to occur solely in a predetermined separate room promoted as the only place where breastfeeding was accepted, thus contributing to uneasiness and insecurity with breastfeeding in a public domain. Finally, women also suggested how breastfeeding-adapted clothes were important in a supportive environment and facilitated public breastfeeding for women.

*Breastfeeding spaces other than those tucked away in the toilets at shopping centres.* [Aus; 117]

### Theme: the obvious right of breastfeeding women and children to take a seat in the public sphere

The three subthemes that make up this theme around the rights of women and children include ‘Zero tolerance for others’ unwanted opinions’; ‘Focus on the needs and rights of the breastfeeding dyad’; and ‘Desexualize breastfeeding and women’s bodies in society’.

#### Subtheme

‘Zero tolerance for others’ unwanted opinions’.

When encountering a breastfeeding woman in the public sphere, a wish for greater respect from the surrounding citizens was expressed. The women emphasized that people in general should not have to defend how they were providing nutrition to their infant. Instead, breastfeeding should be considered as the norm.

*Anyone being rude / derogatory to a mum should be asked to leave the premises.* [Ire; 1538]

Women asked for verbal support or civil courage from other citizens, when they were exposed to derogatory comments.

#### Subtheme

‘Focus on the needs and rights of the breastfeeding dyad’.

The rights of women to choose where and when they feed their baby was paramount to women in our study, a legally accepted place in the public sphere. Otherwise, there is a risk that women feel isolated in their home. Society needs to be inclusive where women and their breastfeeding children can participate in everyday life, not being restricted to separate rooms or places for breastfeeding. Our participants also noted that society has a responsibility to support the mother and child dyad also in breastfeeding and fundamental needs of the child, as stated by the women.

*That the needs of a newborn child regarding nutrition and closeness have to be satisfied irrespective of location. Otherwise, there is a risk that mothers stay at home, isolating themselves.* [Swe; 1020]

#### Subtheme

‘Desexualize breastfeeding and women’s bodies in society’.

The women expressed a desire that their breasts should be recognized for their function in providing breast milk, not as a sexual attribute. They stated that they wanted to “take back” the right to their own body and not have to be subject to awkward glances or adverse stares from members of the public. The participants expressed that the breast should be accepted for its vital function of breastfeeding, not as the main feature of lingerie advertisements. Equality between the sexes was brought up as a factor that impacted the sexualization of the breast.

*Raise boys and educate men that breastfeeding/breasts have no sexual connection.* [Swe; 878]

### Comparison of the most important factors to encourage a culture of public breastfeeding

The subthemes that captured Australian, Swedish and Irish women’s perceptions of what they ranked as the number one most important factor to encourage a breastfeeding culture is presented in Table 3. When comparing the ranking of each of the subthemes, the commonalities were regarding the importance of the visibility of breastfeeding in society. ‘Education of the public’ was the second most highly ranked subtheme across all countries as a suggestion to improve the culture for women breastfeeding in public. The need for the surrounding environment to be inviting was ranked third across all countries. The women from Sweden and Ireland ranked ‘Healthcare professionals support and knowledge regarding breastfeeding’ as the fourth most important issue. Australia women ranked ‘Desexualizing breastfeeding and the female body’ (9%), closely followed by ‘Focusing on the needs and rights of the breastfeeding dyad’ (8%) and ‘Zero tolerance to others’ unwanted opinions’ (6%). Australian women ranked ‘Healthcare professionals support and knowledge regarding breastfeeding’ in seventh place, which was lower than the ranking in the other two countries.

## Discussion

The results of this study illustrate both practical and structural factors supporting a culture of breastfeeding in public.

In all three countries, seeing other women breastfeed in the public sphere was considered most important to encourage a culture where breastfeeding is a ‘normal’ part of life and commonly seen in society. The women wanted breastfeeding to be viewed as something women do to nourish and to be close to their children which aligns with the results of Hoddinott et al. [23] where seeing other women breastfeed could increase the awareness of breastfeeding.

Therefore, providing separate rooms for breastfeeding that are out of sight can negatively affect the public breastfeeding culture [24] and contribute to a feeling of being “hidden” [25]. Similar to Amir [14], who adressed the idea of a middle way between closed-off breastfeeding rooms and a crowded food court, reflections occured about the fact that although separate breastfeeding rooms are appreciated by some women, this can contribute to structural problems to ensure the existence of a specific place where breastfeeding should be performed. To protect the woman and her nursing child, options should be offered to each individual women, to have the right to decide what suits her and her child best. One condition the women reinforced as important to breastfeed in public is the access to a breastfeeding-friendly environment which aligns with the findings of Boyer [26] who describes the importance of multiple adjustable seating, adequate temperature and lighting as well as the opportunity to sit together with others.

Similar to the results regarding HCP providing insufficient and out-of-date advice, former issues about lack of knowledge regarding breastfeeding have been described in research, suggesting that HCP often lack adequate and up-to-date knowledge and therefore offer insufficient and out-of-date advice regarding breastfeeding [27–29]. However, it is not only HCP who need to gain better knowledge about breastfeeding. Our results show that the respondents also perceived a general lack of knowledge amongst the public. The women stated that this could be a contributing factor to why the attitude towards breastfeeding in the public sphere is perceived to be negative. This can be interpreted as a need to provide education for the general public, starting preferably in primary school, as a factor that could be highly important for promoting a better culture regarding public breastfeeding, in accordance with the global Sustainable Development Goals [30] as well as the Convention on the Rights of the Child [3] which states that all parents have the right to adequate education regarding child health care, nutrition and benefits of breastfeeding.

Our findings, that breastfeeding women should have a right to take a place in society is reflected in our second theme, where the rights of women and children must be given a central place in the public sphere. The theme primarily reflects underlying structural factors in society that have influenced the culture around public breastfeeding. The discrimination legislation [7] also points out that consideration must be given to gender-specific conditions and diseases that are more common among women; breastfeeding could fall under this category. In the question of the child’s human rights in this context, the Convention on the Rights of the Child [3] reinforces these by underlining the importance of “. . ensuring the child such protection and care as is necessary for his welfare, taking into account the rights and obligations of his parents. . .“ [sic] (p. 7). The Convention on the Rights of the Child was passed into law in Sweden in 2020.

The women in this study had often attained a high level of education, had a mean age above 32 years, (Sweden 32.7, Australia 32.5 and Ireland 34.9), and came from high income developed countries. Research confirms that highly educated women are those who breastfeed to a greater extent [31–33]. Therefore, our results are not representative of all women, which is a limitation. There are no studies suggesting that highly educated women breastfeed in public to a greater extent than women with lower education. However, these women may have a higher breastfeeding self-efficacy related to higher incidence and confidence [34] in relation to their experience of breastfeeding [35]. This potential explanation could contribute to a greater sense of security when breastfeeding in public. Further research is needed and must also focus upon younger women with lower education levels and women from developing countries. The study’s high response rates and an international focus is a strength as credibility, trustworthiness and transferability are considered related to the qualitative method, and with analysis across three countries involving multiple researchers [36]. It is apparent that women want to have a voice on breastfeeding in public and share their thoughts and perceptions around these. Three high income developed countries provided an opportunity to compare women’s experiences. A limitation of the study is the fact that the results are based on an open-ended question in a survey, which does not provide the same depth as an interview. Therefore, this study can be seen as one way to explore the topic of breastfeeding in public.

## Conclusion

The most important factor cited by women in all three countries to encourage a culture of public breastfeeding was to make breastfeeding more visible in society, followed by higher education of the public and an inviting environment. Women highlighted their obvious right to take a seat in the public sphere when breastfeeding. The common experience that exists today regarding public breastfeeding requires change towards public normalization. Further collaborative research is recommended.

## Data Availability

The datasets generated and analysed during the current study are not publicly available but are available from the corresponding author on reasonable request.

## Abbreviations

HCP: Health care professional
WHO: World health organization
